# Immunologic Effects of Vitamin D on Human Health and Disease

**DOI:** 10.3390/nu12072097

**Published:** 2020-07-15

**Authors:** Nipith Charoenngam, Michael F. Holick

**Affiliations:** 1 Vitamin D, Skin, and Bone Research Laboratory, Section Endocrinology, Diabetes, Nutrition and Weight Management, Department of Medicine, Boston University School of Medicine, 85 E Newton St, M-1013, Boston, MA 01228, USA; ncharoen@bu.edu; 2 Department of Medicine, Faculty of Medicine Siriraj Hospital, Mahidol University, Bangkok 10700, Thailand

**Keywords:** vitamin D, immune function, 25-hydroxyvitamin D, 1,25-dihydroxyvitamin D, immunomodulation, autoimmune disorders, infectious diseases, lymphocytes, monocytes, macrophages, multiple sclerosis, type 1 diabetes, inflammation, endothelial membrane stability

## Abstract

Vitamin D is responsible for regulation of calcium and phosphate metabolism and maintaining a healthy mineralized skeleton. It is also known as an immunomodulatory hormone. Experimental studies have shown that 1,25-dihydroxyvitamin D, the active form of vitamin D, exerts immunologic activities on multiple components of the innate and adaptive immune system as well as endothelial membrane stability. Association between low levels of serum 25-hydroxyvitamin D and increased risk of developing several immune-related diseases and disorders, including psoriasis, type 1 diabetes, multiple sclerosis, rheumatoid arthritis, tuberculosis, sepsis, respiratory infection, and COVID-19, has been observed. Accordingly, a number of clinical trials aiming to determine the efficacy of administration of vitamin D and its metabolites for treatment of these diseases have been conducted with variable outcomes. Interestingly, recent evidence suggests that some individuals might benefit from vitamin D more or less than others as high inter-individual difference in broad gene expression in human peripheral blood mononuclear cells in response to vitamin D supplementation has been observed. Although it is still debatable what level of serum 25-hydroxyvitamin D is optimal, it is advisable to increase vitamin D intake and have sensible sunlight exposure to maintain serum 25-hydroxyvitamin D at least 30 ng/mL (75 nmol/L), and preferably at 40–60 ng/mL (100–150 nmol/L) to achieve the optimal overall health benefits of vitamin D.

## 1. Introduction

**Figure 1:**
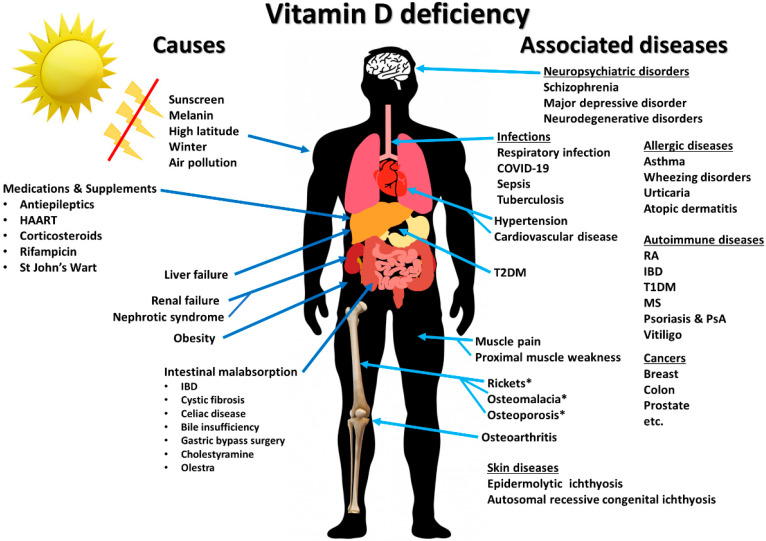
Summary of causes of vitamin D deficiency and diseases and disorders associated with vitamin D deficiency. Abbreviations: HARRT: highly active antiretroviral therapy; IBD: inflammatory bowel diseases; MS: multiple sclerosis; PsA: psoriatic arthritis; T1DM: type 1 diabetes mellitus; T2DM: type 2 diabetes mellitus; RA: rheumatoid arthritis. Reproduced with permission from Holick MF, copyright 2020. ‘*’ denotes diseases that are direct consequences of vitamin D deficiency.

Vitamin D is classically known to regulate calcium and phosphate metabolism. It not only plays an essential role in maintaining healthy mineralized skeleton, but also is an immunomodulatory hormone [1,2]. Both the vitamin D receptor (VDR) and metabolizing enzymes are expressed by various types of immune cells including lymphocytes, monocytes, macrophages, and dendritic cells [3,4]. Experimental studies have shown that vitamin D has significant biologic activities on the innate and adaptive immune systems. Animal studies have demonstrated that administration of vitamin D or its metabolites leads to changes in the occurrence and progression of various immune-related diseases [1,5]. This supports the clinical and epidemiological data that link vitamin D with the incidence and severity of many disorders such as psoriasis, multiple sclerosis, rheumatoid arthritis, type 1 diabetes, and infectious diseases [2] (Figure 1). The purpose of the present review is to provide a high-level summary of the biologic effects of vitamin D on the immune system and the relationship between vitamin D and several types of immune-related diseases and conditions. This review also aims to give some perspective about the heterogeneity of evidence on the impact of vitamin D on prevention and treatment of immune-related diseases and to introduce the concept individual responsiveness to vitamin D as a potential explanation for such heterogeneity.

## 2. Physiology of Vitamin D

### 2.1. Sources, Synthesis, and Metabolism of Vitamin D

Human gets vitamin D from sunlight, diet, and supplements. There are two major forms of vitamin D: vitamin D_2_ and vitamin D_3_. Vitamin D_2_ is synthesized from ergosterol and found in yeast, sun dried and ultraviolet irradiated mushrooms, and plants. Vitamin D_3_ is synthesized endogenously from 7-dehydrocholesterol in the skin and found naturally in cod liver oil and oily fish. After entering the circulation, vitamin D (D represents either or both vitamin D_2_ and vitamin D_3_) is metabolized by the vitamin D-25-hydroxylase (CYP2R1) in the liver to 25-hydroxyvitamin D [25(OH)D]. 25(OH)D is further metabolized by the enzyme 25-hydroxyvitamin D-1α-hydroxylase (CYP27B1) to the active form, 1,25-dihydroxyvitamin D [1,25(OH)_2_D] [2,6]. 1,25(OH)_2_D exerts its physiologic functions in the target tissue by binding to the vitamin D receptor (VDR) in the nucleus where it leads to up- or down-regulation of a multitude of genes [7]. It should be noted that the main site of conversion of 25(OH)D into the systemically bioavailable 1,25(OH)_2_D is the kidneys. CYP27B1 is also expressed by many other tissues including activated macrophages, parathyroid glands, microglia, breast, colon, and keratinocytes where 1,25(OH)_2_D is produced and exerts its autocrine and paracrine functions [1,4,5].

### 2.2. Skeletal Effects of Vitamin D

1,25(OH)_2_D regulates calcium and phosphate homeostasis by acting on the small intestine, kidneys, and bone. It promotes bone mineralization passively by inducing intestinal calcium and phosphate absorption and renal tubular calcium reabsorption that help maintain an adequate calcium-phosphate product that crystallizes in the collagen matrix. 1,25(OH)_2_D also has direct effects on the bone, including inducing the expression of osteocalcin, the major non-collagenous protein in the skeleton, and stimulating receptor activator of nuclear factor kappa-B (RANK)-dependent bone resorption [2,6]. In addition, 1,25(OH)_2_D forms an endocrine system together with parathyroid hormone (PTH) and fibroblast growth factor 23 (FGF23) to regulate calcium and phosphate homeostasis [8]. As a part of negative feedback loops, 1,25(OH)_2_D directly inhibits PTH production, leading to a decrease in bone resorption and increased urinary calcium excretion, and induces FGF23 production by the osteocytes, leading to an increase in urinary phosphate excretion [6,8,9].

### 2.3. Healthy Serum Vitamin D and 25-Hydroxyvitamin D Levels

It is still controversial how much vitamin D is needed, how it should be given, i.e., daily versus weekly or monthly (bolus doses), and what level of serum 25(OH)D is optimal for immune health and overall health benefits [10,11]. It is also unknown whether maintenance of serum vitamin D itself has its own effect on modulating immune function. However, historical evidence suggests that our hunter gatherer forefathers maintained their circulating vitamin D levels in the range of 10–50 ng/mL (25–125 nmol/L). Indigenous populations such as Maasai herders and Hadza tribesmen were found to have serum 25(OH)D levels in the range of 40–60 ng/mL (100–150 nmol/L) [12,13,14]. These levels are consistent with those reported in populational studies to be associated with the lowest risk of several types of cancers, cardiovascular diseases, autoimmune diseases, and all-cause mortality [2,10,14,15]. To maintain these blood levels with minimal sunlight exposure, a person would require ingestion of 4000–6000 IUs of vitamin D daily, which would maintain serum vitamin D levels in the range of 20–40 ng/mL (50–100 nmol/L) and serum 25(OH)D levels in the range of 40–60 ng/mL (50–100 nmol/L) [11]. The recommended dosage for vitamin D intake by the Endocrine Society Guidelines on Vitamin D for treatment and prevention for vitamin D deficiency is shown in Table 1.

## 3. Effects of Vitamin D on Innate Immunity

### 3.1. Macrophages and Monocytes

**Figure 2:**
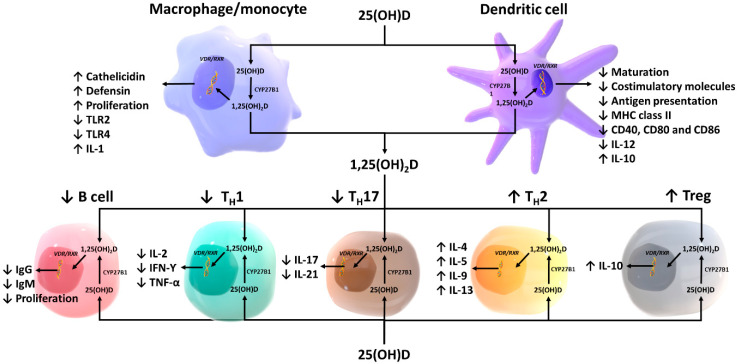
Schematic representation of paracrine and intracrine function of vitamin D and its metabolites and actions of 1,25-dihydroxyvitamin D on the innate and adaptive immune systems. Abbreviation: 1,25(OH)_2_D: 1,25-dihydroxyvitamin D; 25(OH)D: 25-hydroxyvitamin D, IFN-Ƴ: interferon- Ƴ; IL: interleukin; MHC: membrane histocompatibility complex, T_H_1: T helper 1; T_H_2: T helper 2; T_H_17: T helper 17; Treg: regulatory T cell, TNF-α: Tumor necrosis factor- α; TLR2: toll-like receptor 2; TLR4: toll-like receptor 4. Reproduced with permission from Holick MF, copyright 2020.

Historical evidence that links vitamin D with innate immunity came from the reports in the mid-1800s and early 1900s prior to the antibiotic era that vitamin D_3_-rich cod liver oil and sunlight exposure were used for treatment of tuberculosis (TB) [16]. Later, subsequent studies have identified the explanation for the therapeutic effects of cod liver oil and sunlight. In the presence of infection, activated macrophages and monocytes, induced by toll-like receptor signaling and exposure to inflammatory cytokines such as interferon-Ƴ (IFN-Ƴ), strongly express CYP27B1 which converts 25(OH)D into 1,25(OH)_2_D [17]. Then, 1,25(OH)_2_D enhances antimicrobial activities of macrophages and monocytes in an autocrine fashion via VDR-RXR signaling, which, in turn, stimulates the production of endogenous antimicrobial cathelicidin LL-37 [1,2,5,17,18]. Cathelicidin acts against invading bacteria and fungi by destabilizing microbial membranes [19,20]. It also exhibits direct antiviral activities against many respiratory viruses by disrupting viral envelopes and altering viability of host target cells [20,21,22]. This process is especially robust in granulomatous inflammation such as TB, fungal infections, sarcoidosis, and some lymphomas. The macrophage production of 1,25(OH)_2_D not only is for the purpose of upregulating the production of cathelicidin LL-37 but also produces it so that it can exit the cell and influence nearby lymphocyte function [2] (Figure 2). However, an unintended consequence of this paracrine function is that the macrophages produce an excess amount of 1,25(OH)_2_D that enters the circulation and an unregulated fashion stimulates intestinal calcium absorption and bone calcium mobilization resulting in hypercalciuria and hypercalcemia [23,24]. This is more likely to occur when circulating levels of 25(OH)D are above 30 ng/mL (75 nmol/L) [11], which is the explanation for why patients with granulomatous disorders including sarcoidosis develop hypercalcemia during the summer [25].

### 3.2. Antigen-Presenting Cells and Natural Killer Cells

1,25(OH)_2_D modulates the differentiation and functions of antigen-presenting cells by inducing them to become more immature and tolerogenic, characterized by a decrease in the expression of major histocompatibility complex (MHC) class II and co-stimulatory molecules on the cell surface [26,27,28,29] (Figure 2). This results in a decrease in antigen presentation and production of interleukin-12 (IL-12), and an increase in production of IL-10, a tolerogenic cytokine [29,30,31]. 1,25(OH)_2_D is also shown to suppress the expression of toll-like receptors on the monocytes and inhibit the production of some inflammatory cytokines such as IL-2, IL-6, and IL-17 [5,32,33]. In addition, experimental studies have suggested that differentiation and function of natural killer (NK) cells can be modulated by 1,25(OH)_2_D treatment. However, whether 1,25(OH)_2_D induces or inhibits NK cell function is still unclear as data regarding the influence of 1,25(OH)_2_D on NK cells are inconsistent [34,35,36]. 

### 3.3. Endothelial Function and Vascular Permeability

A number of experimental studies have shown that vitamin D and its metabolites modulate endothelial function and vascular permeability via multiple genomic and extra-genomic pathways. For instance, Gibson et al. [37] demonstrated in the primary dermal human microvascular endothelial cell model that vitamin D_3_, 25(OH)D_3_ and 1,25(OH)_2_D_3_ non-genomically stabilized vascular endothelium, and that vitamin D_3_, normally circulating at about 100 times higher level than 1,25(OH)_2_D_3_, was at least 10 times more potent than 1,25(OH)_2_D_3_ and more than thousand times more potent than 25(OH)D_3_ in stabilizing the endothelium. Studies have also shown that 1,25(OH)_2_D_3_ is a transcriptional regulator of endothelial nitric oxide synthase (eNOS), causing up-regulation of eNOS gene expression and therefore increased endothelial production of nitric oxide [38,39]. Interestingly, Molinari et al. [40] observed that the effect of 1,25(OH)_2_D_3_ on endothelial nitric oxide production occurred most robustly within one minute after administrating the compound, implying that the action of 1,25(OH)_2_D_3_ was non-genomic. Activation of VDR by 1,25(OH)_2_D at the endothelial cell membrane is shown to increase eNOS activity via intracellular second messenger pathways including adenylyl cyclase/cyclic adenosine monophosphate (AC/cAMP) and inositol trisphosphate/diacylglycerol (IP_3_/DAG) pathways, which result in increased intracellular calcium concentration. Activation of VDR also activates eNOS via the phosphoinositide 3-kinase/protein kinase b (PI3K/Akt) pathway that triggers phosphorylation of serine-1779 on eNOS [41]. Furthermore, Cuenca et al. [42] showed in a uremic rat model using the immunofluorescence technique that 1,25(OH)_2_D_2_ promoted vascular endothelial-cadherin-based cell–cell junctions and inhibited F-actin stress fiber organization, thereby preventing the formation of endothelial intracellular gaps and attenuating endothelial damage in the presence of chronic kidney disease. Taken together, it is evident that vitamin D and its metabolites exert pleiotropic effects on the vascular endothelium that are protective against vascular dysfunction and tissue injury as a result of local and systemic inflammation.

### 3.4. Intestinal Epithelium and Paneth Cells

Multiple studies have shown that vitamin D plays a role in maintaining gut integrity and intestinal homeostasis between host and gut microbiota. Vitamin D signaling is shown to increase the viability of intestinal epithelial cells and and alleviate intestinal epithelial damages from bacterial lipopolysaccharide [43,44]. It promotes mucosal barrier function by enhancing the expression of intracellular pathogen recognition proteins and epithelial membrane junction proteins [45,46]. Moreover, 1,25(OH)_2_D induces the production and secretion of antimicrobial peptides by the intestinal epithelial cells, Paneth cells, and intraepithelial lymphocytes [47,48]. These result in limitation of gut bacterial translocation into the interstitium and maintenance of intestinal homeostasis, which are believed to involve in the pathogenesis of multiple autoinflammatory and metabolic disorders [49,50].

## 4. Effects of Vitamin D on Adaptive Immunity

### 4.1. T lymphocytes

It was originally observed that a human monocyte cell line U937, clonal human T cells, and peripheral human blood mononuclear cells express VDR [51,52]. Further analysis revealed that peripheral blood monocytes express VDR but that resting T lymphocytes did not. When resting T lymphocytes were stimulated with phytohemagglutinin, they became activated and with the activation, T cells expressed the VDR [51,52]. Moreover, activated T lymphocytes are known to express CYP27B1 that mediates local conversion of 25(OH)D into 1,25(OH)_2_D which is believed to stimulate intracrine activation of VDR [53,54] (Figure 2).

In general, 1,25(OH)_2_D produced locally by monocytes/macrophages results in a dramatic shift of immune status from proinflammatory state to tolerogenic state. 1,25(OH)_2_D suppresses the proliferation of T lymphocytes, and modulates cytokine production and differentiation with diverse effects on different subgroups of T lymphocytes [55]. It promotes a shift from T_H_1 and T_H_17 to T_H_2 immune profile by suppressing the expression of T_H_1 (IL-2, IFN-Ƴ, TNF-α) and T_H_17 (IL-17, IL-21) cytokines while inducing the expression of T_H_2 cytokines (IL-4, IL-5, IL-9, IL-13) [56,57,58]. 1,25(OH)_2_D can also promote differentiation of regulatory T cells (Treg) both directly and indirectly via its interaction with antigen-presenting cells, resulting in a suppression of proinflammatory state [59,60]. This is believed to be one of the explanations by which vitamin D could exert protective effects against autoimmune diseases. 

Similar to T helper cells, cytotoxic T lymphocytes (CTL) express both CYP27B1 and VDR, and upregulation of VDR can be observed in response to infection as well as mitogen stimulation, suggesting a coordinate regulation of VDR signaling pathway and CTL responses [61,62]. Studies have shown that decreased CD4/CD8 ratio, an indicator of increased immune activation, was associated with low levels of 25(OH)D [63] and that giving 5000–10,000 IUs of vitamin D_3_ was associated with an increase in CD4/CD8 ratio, reflecting immune suppression [64,65]. However, little is known about the direct influence of vitamin D on CTL. The effects of 1,25(OH)_2_D on differentiation, proliferation, and functions of CTL are likely mediated by both direct intracrine activation of VDR and alteration of cytokines signaling via T helper cells and antigen-presenting cells [55,62].

### 4.2. B lymphocytes

Inactive B lymphocytes have no VDR, and only when they become activated to proliferate by mitogens do they upregulate their VDR expression [66] (Figure 2). Initially, it was found that 1,25(OH)_2_D inhibited immunoglobulin synthesis and therefore could potentially be detrimental to the immune system. However, a variety of studies have demonstrated that just as 1,25(OH)_2_D modulates T cell function so too does it regulate B-cell activity. When in a hyperactive state, 1,25(OH)_2_D appears to dampen the immunoglobulin immune response by a variety of mechanisms. It inhibits plasma cell formation and inducing apoptosis of both activated B cells and plasma cells [67,68]. It also inhibits cytokine-mediated B-cell activation by acting on T-helper cells, directly promotes B-cell anti-inflammatory cytokines production (IL-10, CCR10) and suppresses the differentiation from mature B cells to plasma cells and class-switched memory B cells [66,69,70]. It is believed that, by controlling B-cell activity and B-cell transformation into the plasma cells, 1,25(OH)_2_D helps reduce autoantibody production, thereby reducing risk for antibody-mediated autoimmune disorders such as systemic lupus erythematosus [66,71]. It has also been suggested that lymphocytes also have the capacity to generate 1,25(OH)_2_D. What is known is that only B lymphocytes can express CYP27B1 and that 25(OH)D in vitro at 25 times the level of 1,25(OH)_2_D can cause similar biologic responses as 1,25(OH)_2_D in B lymphocytes [66]. However, there has not been any direct demonstration that B lymphocytes can produce 1,25(OH)_2_D.

## 5. Vitamin D and Immune-Related Diseases

### 5.1. Psoriasis

1,25(OH)_2_D was well-documented to be a very effective hormone in inhibiting proliferation and inducing terminal differentiation of a wide variety of cultured malignant cells including prostate cancer, colon cancer, breast cancer, and leukemic cells [2]. However, 1,25(OH)_2_D and its analogs have never been successfully developed for treating any cancer. An attempt was made to treat preleukemia; however, not only did 1,25(OH)_2_D_3_ cause undesirable hypercalcemia, but also ultimately the leukemic cells became resistant to the antiproliferative activity [72]. Psoriasis, however, is a non-malignant hyperproliferative disorder of the skin that is a chronic inflammatory disorder affecting 2–3% of global population [73]. It has been well documented that the epidermis is the major site for the cutaneous production of vitamin D_3_ [74]. It was also recognized that keratinocytes express VDR and 1,25(OH)_2_D was extremely effective at physiologic concentrations not only in inhibiting cultured keratinocyte proliferation, but also in inducing their terminal differentiation [75,76]. These observations gave rise to the idea that 1,25(OH)_2_D could be developed for treating this hyperproliferative skin disorder [77]. 

It had been previously observed that cultured dermal fibroblasts from psoriatic patients had a partial resistance to the antiproliferative activity of 1,25(OH)_2_D [78]. Further studies revealed that only about 20% of patients with psoriasis had fibroblasts with a partial resistance to the antiproliferative activity of 1,25(OH)_2_D_3._ It was observed that cultured keratinocytes from psoriasis patients responded to the antiproliferative and pro-differentiating activity of 1,25(OH)_2_D_3_ as well as cultured keratinocytes obtained from neonatal foreskins [79]. This led to a pilot study to evaluate topical and oral 1,25(OH)_2_D_3_ to determine its clinical effectiveness [79]. However, there was concern about the hypercalcemic potential of clinically using 1,25(OH)_2_D_3_. This prompted the development of an analog of 1,25(OH)_2_D_3_ that was considered to have less calcemic activity. This analog, calcipotriene (50 mcg/g), was found to be reasonably effective when topically applied for treating psoriasis [80]. No significant hypercalcemia was observed unless large body surface was treated. However, the major side effect was skin irritation that occurred mainly on the face and therefore it was not recommended for treating psoriasis on the face and other sensitive skin areas [80]. 1,25(OH)_2_D_3_ was demonstrated to be effective at 15 mcg/g and also was found to be safe and effective and did not cause skin irritation. 1,25(OH)_2_D_3_ was finally developed as a topical treatment and formulated as 3 mcg/g [77]. Other analogs have since been developed and are available for treating psoriasis skin lesions that usually affect less than 10% of the body surface. Oral 1,25(OH)_2_D_3_ was also found to be safe and effective but was not developed as a pharmaceutical because of concerns about hypercalcemia.

It is now recognized that there is a strong immunologic component to psoriasis. The hyperproliferation of keratinocytes is associated with T_H_1, T_H_17, and T_H_22 inflammatory response to self-antigen [81]. 1,25(OH)_2_D exerts inhibitory effects against the inflammatory activity associated with psoriasis not only by suppressing dendritic cell’s differentiation, chemotaxis, and antigen presentation, but also by inhibiting the production of several pro-inflammatory cytokines such as IL-1β, IL-6, IL-8, and TNF-α [82]. 

Vitamin D deficiency, defined by serum 25(OH)D <20 ng/mL (50 nmol/L), has been reported as one of the independent risk factors for psoriasis. Multiple observational studies found a higher prevalence of vitamin D deficiency among patients with psoriasis compared with the general population, even after adjusting for confounders in a multivariate analysis [82,83]. The likely explanation is that patients with psoriasis are less likely to expose their skin to sunlight which is a major source of vitamin D. However, correcting vitamin D deficiency may be beneficial. 

Multiple clinical trials have attempted to investigate the influence of different forms of vitamin D supplementation on treatment of psoriasis, yielding varying results. A six-month study of psoriasis patients who received 60,000 IUs of vitamin D_2_ once every 2 weeks not only showed significant improvement in their PASI (psoriasis area and severity index) score but also demonstrated a direct association with improvement in blood levels of 25(OH)D [84]. Another pilot study giving daily 35,000 IUs of vitamin D_3_ to 9 patients with psoriasis and 16 patients with vitiligo who were vitamin D-deficient or insufficient for six months demonstrated a significant improvement in psoriasis area and severity index (PASI) in all psoriatic patients and 25–75% repigmentation in 14 of 16 vitiligo patients, without development of any complications suggestive of vitamin D toxicity including hypercalcemia, kidney stones, nephrocalcinosis, or hypercalciuria [85]. On the other hand, a one-year study giving 100,000 IUs per month of vitamin D_3_ or placebo to psoriatic patients for one year did not demonstrate a direct benefit of vitamin D supplementation on disease activity [86]. The study however revealed that the severity of psoriasis was less for those with higher blood levels of 25(OH)D [86]. Based on the evidence from these studies, it is advisable to test and treat patients with psoriasis for vitamin D deficiency in order to maintain their serum 25(OH)D levels in a preferred range of 40–60 ng/mL (100–150 nmol/L) [11]. 

In addition, oral administration of 1,25(OH)_2_D_3_ has been shown to have some therapeutic effects on psoriasis. A long-term follow-up study demonstrated the efficacy and safety of oral calcitriol as a potential treatment of psoriasis. Of the 85 patients included in that study that received oral calcitriol for 36 months, 88.0% had some improvement in their disease, whereas 26.5%, 26.3%, and 25.3% had complete, moderate, and slight improvement in their disease, respectively. Serum calcium concentrations and 24-h urinary calcium excretion increased by 3.9% and 148.2%, respectively, but were not outside the normal range. Bone mineral density of these patients remained unchanged. A very important consideration for the use of orally administration is the 1,25(OH)_2_D_3_ dosing technique. To avoid its effects on enhancing dietary calcium absorption, it is very important to provide 1,25(OH)_2_D_3_ at night time. Perez et al. [87] showed that as a result of this dosing technique, doses of 2–4 µg/night of 1,25(OH)_2_D_3_ were well tolerated by psoriatic patients. Ezquerra et al. demonstrated that a combination of acitretin and oral 1,25(OH)_2_D_3_ resulted in a faster reduction of PASI score in patients of chronic plaque psoriasis than acitretin alone [88]. Given the small sample sizes in each study, more studies with a larger number of participants are warranted to demonstrate the efficacy of oral 1,25(OH)_2_D for treatment of psoriasis.

### 5.2. Type 1 Diabetes

Finland has the highest incidence of type 1 diabetes in the world [89]. Many theories have been proposed to explain the epidemic such as exposure to certain toxic substances [90] and coxsackievirus B infections that trigger the development of autoimmunity [91]. It is however likely that this can be explained by the lack of sunlight exposure resulting in a high rate of vitamin D deficiency in Finnish population especially in northern Finland. Finns, similar Norwegians living in the far north, are unable to produce vitamin D_3_ in their skin from sun exposure for more than half a year during the winter and early spring and late fall [13,92,93]. This is also supported by the previous report that type 1 diabetes is more common in countries with high latitude and short daytime period [94].

Although not completely understood, type 1 diabetes is believed to be mediated by the development of by autoantibody as well as autoreactive T_H_1 and CTL, causing immune-associated destruction of insulin-producing pancreatic β cells [95,96]. Administration of 1,25(OH)_2_D_3_ was found to enhance Treg and inhibit T_H_1, leading to a reduction in the incidence of type 1 diabetes in the non-obese diabetic mouse model [60]. In addition, 1,25(OH)_2_D_3_ promoted insulin secretion directly by its interaction with VDR in the pancreatic β cells [97]. These mechanisms help explain how vitamin D has a potential protective and therapeutic role in reducing risk for developing type 1 diabetes and support observational studies that increasing vitamin D intake for children was associated with a lower risk for developing type 1 diabetes [98].

The EURODIAB multicenter case-control study in 825 type 1 diabetes patients and 2335 controls revealed that vitamin D supplementation of any dose was associated with a reduced risk of type 1 diabetes (odds ratio, 0.67; 95%CI: 0.53–0.86) [98]. A Finnish cohort study of 10,366 children showed that those who were given daily 2000 IUs of vitamin D_3_ during their first year of life had a 88% reduction in the risk of developing type 1 diabetes (relative risk, 0.22; 95% CI: 0.05–0.89) [99].

Evidence from randomized controlled trials (RCTs) has suggested that vitamin D supplementation in the form of 1α-hydroxyvitamin D_3_ [1α(OH)D_3_] and vitamin D_3_ appeared to be of benefit in the treatment of type 1 diabetes. These treated patients had a significant reduction in daily insulin dose as well as an increase in fasting and stimulated C-peptide levels [100,101,102,103]. On the other hand, studies that evaluated the benefit of maternal vitamin D supplement for prevention of type 1 diabetes in offspring were unable to demonstrate the association [104]. This is not unexpected especially if this autoimmune disorder is initiated during childhood and not in utero.

In summary, increasing vitamin D intake in early childhood to maintain serum 25(OH)D in the optimal range seems to be protective against the development of type 1 diabetes. Supplementation of vitamin D, although not curative, tends to help control the disease activity. However, there is still no evidence for the long-term effects of vitamin D supplementation on morbidities and mortalities in type 1 diabetes patients.

### 5.3. Multiple Sclerosis

The prevalence of multiple sclerosis (MS) is higher in countries with higher latitudes where people are more susceptible to vitamin D deficiency similar to what has been observed in type 1 diabetes [105]. Living below the latitude of 35° for the first 10 years of life is associated with approximately 50% lower risk of developing MS [106]. Munger et al. reported in a prospective nested case-control study of 148 MS patients and 296 controls that the risk of MS decreased by 41% for every 20 ng/mL (50 nmol/L) increase in serum 25(OH)D levels above 24 ng/mL (60 nmol/L) (odds ratio, 0.59; 95% CI, 0.36–0.97) [107]. The same group also showed that women who ingested more than 400 IUs of vitamin D per day had a 41% decreased risk of developing MS [108]. It is therefore believed that vitamin D deficiency plays a role in the development of dysregulated T helper cells, CTL, NK cells, B cells resulting in autoinflammation of the central nervous system that damages neurons and oligodendrocytes seen in MS [109,110]. 

Individuals carrying certain human leukocyte antigen (HLA) alleles such as HLA-DRB1*1501 have a significantly higher risk of developing MS [111]. Interestingly, vitamin D response elements have been identified in the promoter region of the *HLA-DRB1* gene, and its expression can be altered by activation of VDR by 1,25(OH)_2_D, strengthening the link between vitamin D and MS [112,113].

It is conceivable that many actions of 1,25(OH)_2_D on the immune system are similar to mechanisms described for interferon-beta, an immunomodulatory agent used for treatment of MS, implying the possible therapeutic role of vitamin D in MS. Although the results from existing RCTs are conflicting, some studies showed the significant benefits of high-dose vitamin D supplement (up to 14,000 IUs/day) alone or as an add-on therapy in decreasing relapse rate and improving inflammatory markers and MRI findings in MS patients [114]. Most of the existing clinical trials included relatively small number of patients, and dosages of vitamin D given for treatment vary significantly across the studies.

**Figure 3:**
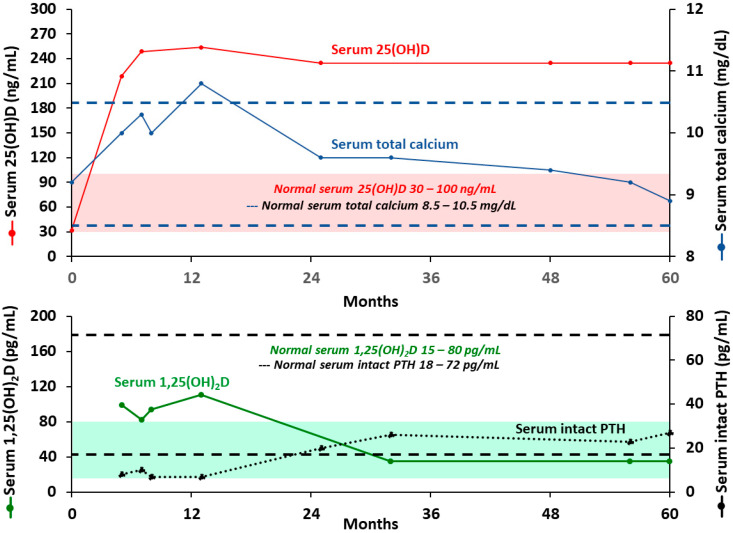
Biochemical measurements of a 52-year-old multiple sclerosis female who received 40,000 international units per day of vitamin D3 for 5 years. Abbreviation: 1,25(OH)2D: 1,25-dihydroxyvitamin D; 25(OH)D: 25-hydroxyvitamin D; PTH: parathyroid hormone. Note: Red solid line represents serum 25-hydroxyvitamin D levels. Blue solid line represents serum total calcium levels. Green solid line represents serum 1,25-dihydroxyvitamin D levels (15–80 pg/mL). Black dotted line represents serum intact parathyroid hormone levels (18–72 pg/mL). Red highlight represents normal range for serum 25-hydroxytamin D (30–100 ng/mL/75–250 nmol/L). Green highlight represents normal range for serum 1,25-dihydroxyvitamin D (15–80 pg/mL). Blue dashed line represents normal range for serum total calcium (8.5–10.5 mg/dL). Black dashed line represents normal range for serum intact parathyroid hormone (18–72 pg/mL). Reproduced with permission from Holick MF, copyright 2020.

**Figure 4:**
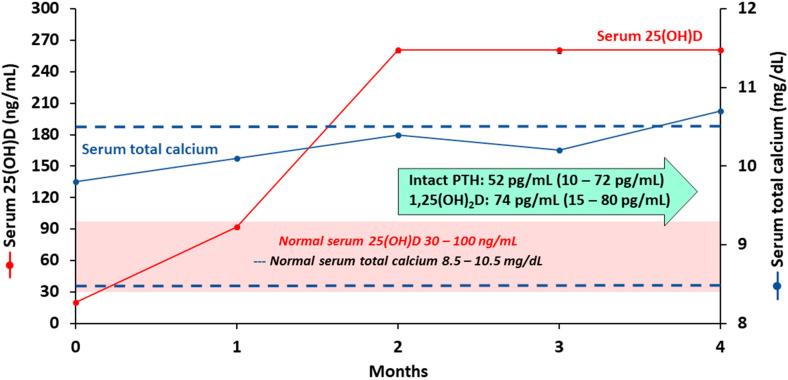
Biochemical measurements of a 32-year-old multiple sclerosis male who received 54,000 international units per day of vitamin D_3_ for 4 months. Abbreviation: 1,25(OH)_2_D: 1,25-dihydroxyvitamin D; 25(OH)D: 25-hydroxyvitamin D; PTH: parathyroid hormone. Note: Red solid line represents serum 25-hydroxyvitamin D levels. Blue solid line represents serum total calcium levels. Red highlight represents normal range for serum 25-hydroxytamin D (30–100 ng/mL/75–250 nmol/L). Blue dashed line represents normal range for serum total calcium (8.5–10.5 mg/dL). Normal range for serum intact parathyroid hormone is 10–72 pg/mL. Normal range for serum 1,25-dihydroxyvitamin D is 15–80 pg/mL. Reproduced with permission from Holick MF, copyright 2020.

Coimbra’s clinical research program in Brazil has been conducting studies with extremely high doses of vitamin D_3_ for treating a variety of autoimmune disorders including among others psoriasis, vitiligo, and multiple sclerosis [85]. The authors’ (MFH) personal clinical experience that treatment with a very high dose of vitamin D supplementation (50,000 IUs/day or 1000 IUs/kg/day) to increase serum 25(OH)D level to 200–300 ng/mL (500–750 nmol/L) was found to be remarkably effective in controlling and/or improving symptoms and improving MRI findings in five MS patients who either failed to respond to or refused conventional MS therapy. The risk of hypercalcemia and hypercalciuria was minimized by advising the patients to strictly follow a zero calcium diet. This required complete elimination of all dairy products and any other foods that contain significant amounts of calcium. A 52-year-old female MS patient was treated with 40,000 IUs/day (1000 IUs/kg/day) of vitamin D_3_ for five years which was found to improve her neurological symptoms. She was advised to avoid all dietary sources of calcium. Serum 25(OH)D was maintained at approximately 250 ng/mL (625 nmol/L) and her total calcium levels transiently increased at approximately 12 months. A review of her diet had revealed that she was eating some vegetables that contained a significant amount of calcium. This supports that calcium was eliminated from her diet and her serum calcium returned to normal levels and have been maintained in a normal range for 5 years. During the first year, serum PTH levels were in the low normal range and serum 1,25(OH)_2_D levels were above the normal range. After the change in her diet both returned into the normal range and have been maintained in the normal range (Figure 3). No hypercalciuria, kidney stones, or nephrocalcinosis was observed. Figure 4 demonstrates changes in serum levels of calcium and calciotropic hormones in a 32-year-old male who refused conventional therapy and was given 54,000 IUs vitamin D_3_ daily. He was able to rapidly increase his circulating level of 25(OH)D and reach a plateau of approximately 250 ng/mL (625 nmol/L) within 2 months. This has been maintained for 4 months and the serum PTH and 1,25(OH)_2_D remained normal as well as his 24-h urine calcium excretion. 

To date, there is no RCT that investigate the efficacy and safety of this massive dose of vitamin D supplement (1000 IUs/kg/day) for MS treatment. Supplementation of lower doses of vitamin D (up to 14,000 IUs/day) appears to have some benefits in controlling the disease activity although limited evidence. What is known is that maintaining sufficient vitamin D intake and serum 25(OH)D level in a healthy range may reduce the risk of developing MS. Further studies are required before this treatment strategy can be implemented in the general clinical practice. 

### 5.4. Inflammatory Bowel Diseases

It is well recognized that patients with inflammatory bowel diseases (IBD) are more prone to vitamin D deficiency, causing them to have a higher risk of osteomalacia, osteoporosis, and fragility fractures [115,116,117,118]. This is because they are unable to efficiently form micelles and chylomicron to absorb vitamin D in their gastrointestinal tract [115,116]. Therefore, these patients should be screened for vitamin D deficiency and treated with a higher dose of vitamin D to achieve a normal serum 25(OH)D level of at least 30 ng/mL (75 nmol/L) [11]. Emerging evidence suggests that the relationship between vitamin D status and IBD could be bidirectional. Data from two prospective Nurses’ Health Studies showed that nurses living in lower latitudes had a consistently lower risk of developing IBD than those in higher latitudes [119]. These observations were supported by a prospective cohort study of 72,719 women enrolled in the Nurses’ Health Study showing that the highest quartile of predicted serum levels of 25(OH)D was associated with 46% reduced risk of Crohn’s disease (CD) and 35% reduced risk of ulcerative colitis (UC) [120].

The pathogenesis of IBD involves a combination of dysfunctional innate and adaptive immunity, defective intestinal epithelial barrier, and imbalanced intestinal microbiota, causing chronic relapsing inflammatory disorder of the intestine [121,122]. CD is thought to be mainly driven by a T_H_1 response, while UC is associated with a T_H_2 response [122,123]. T_H_17 cells are also involved in the inflammatory response in both CD and UC [122,124]. Multiple studies reported that 1,25(OH)_2_D_3_ not only modulates T cell activity by promoting Treg and inhibiting T_H_1 and T_H_17 responses, but also maintains integrity of the intestinal mucosal barrier by enhancing the expression of epithelial membrane junction proteins and intracellular pathogen recognition proteins, and inducing the production of antibacterial substances such as angiogenin, cathelicidin, and defensin by the intestinal epithelial cells, Paneth cells, and intraepithelial lymphocytes [45,46,47,125,126]. 

A meta-analysis of 18 RCTs demonstrated that vitamin D supplementation in patients with IBD is associated with decreased relapse rate, supporting a therapeutic role of vitamin D as an adjunctive treatment of IBD [127]. Moreover, a recent pilot clinical trial showed that giving 380,000 IU of orally administered 25(OH)D supplementation to patients with CD increased the abundance of potential beneficial bacterial strains. [128]. Although this observation remains to be confirmed by a larger clinical trial, a RCT of healthy adults who were vitamin D deficient and received either 600, 4000, or 10,000 IUs daily for 6 months had a similar dose-dependent improvement in their gut microbiota towards genera associated with increased expansion of Treg and lower inflammatory burden [129]. Thus, improvement in the vitamin D status of patients with IBD is warranted not only for modulating the immune response but also improving their gut microbiota.

In summary, patients with IBD could not effectively absorb vitamin D and therefore require a 2–3 times higher dose of vitamin D supplementation to achieve normal serum 25(OH)D levels. Adequate supplementation of vitamin D in IBD is not only required to reduce the risk of osteoporosis, osteomalacia, and fragility fracture, but also considered as an adjunctive immunomodulatory agent that has been shown to improve the disease activity.

### 5.5. Rheumatoid Arthritis

Low levels of serum 25(OH)D have been shown in multiple studies to be associated with an increased risk of RA [130,131]. Merlino et al. [130] showed in a prospective cohort study that women with highest tertile of vitamin D intake had a lower risk for developing RA by 33% compared with the lowest tertile. Some studies also reported the association between low serum 25(OH)D levels and higher disease activity in RA patients [131,132,133]. Although the association could simply be explained the fact that these patients tend to have limited physical outdoor activities and sunlight exposure, vitamin D and its metabolites are believed to have a therapeutic activity against RA based on the immunologic activities of 1,25(OH)_2_D that suppress T_H_1 and T_H_17 responses and enhance Treg activity [134]. Overexpression of T_H_1 and T_H_17 as well as dysfunctional Treg play a crucial role in triggering chronic synovial inflammation and symmetrical polyarthritis seen in RA [135,136,137]. 

The efficacy of vitamin D and its metabolites as an adjunctive treatment for RA is heterogeneous across the clinical trials. Gopinath et al. [138] demonstrated in a RCT that giving 500 IUs of vitamin D_3_ daily along with disease-modifying anti-rheumatic drugs (DMARDs) and calcium to RA patients lead to a significantly higher pain relief than those receiving DMARDs and calcium alone. Another study by Li et al. [139] that randomized RA patients to receive 22-oxa-1,25(OH)_2_D_3_ or 1,25(OH)_2_D_3_ or placebo observed significantly decreased swollen joints and improved Health Assessment Questionnaire Disease Activity Index scores in those receiving 22-oxa-1,25(OH)_2_D and those receiving 1,25(OH)_2_D compared with the placebo group. However, the doses of 1,25(OH)_2_D_3_ and 22-oxa-1,25(OH)_2_D_3_ used in the study were reported to be 1250 µg per week, which was about 1000 times higher than the therapeutic dose of these compounds and would likely cause toxicity, thus indicating that there might be some type of error, possibly a decimal point error [139]. Other studies that used vitamin D_2_ [140], vitamin D_3_ [141,142], or 1α(OH)D_3_ [143] in the treatment arm failed to demonstrate the efficacy of the intervention.

Taken together, there is convincing evidence that increasing vitamin D intake to maintain serum 25(OH)D levels in a preferred range of 40–60 ng/mL (100–150 nmol/L) may reduce the risk of RA. However, there is still not enough evidence to justify whether vitamin D supplementation in any form can improve the outcomes of RA.

### 5.6. Tuberculosis

In the early 1900s, Finsen made an enlightening observation that exposure to sunlight dramatically improved cutaneous TB infection (lupus vulgaris) and received the Nobel Prize in 1903 for his discovery. This resulted in the use of solariums as an effective treatment for TB [13]. Nowadays, tuberculosis continues to be a major public health problem that is a leading cause of morbidity and mortality in many developing countries [144]. Latent TB is the condition in which host immune response is able to form granuloma to engulf the mycobacterium in an attempt to control its proliferation. Once the granuloma fails to limit mycobacterial proliferation, patients become symptomatic and are diagnosed with active TB [145]. Vitamin D plays an essential role in combating TB infection. Activated macrophages and monocytes in response to antigen exposure locally produce 1,25(OH)_2_D which then induces the production of cathelicidin, an antibacterial peptide responsible for killing infectious agents like *Mycobacterium tuberculosis* [1,2,5,17]. 

Multiple studies have reported low levels of serum 25(OH)D in patients with active TB. In a nested case-control study by Aibana et al., individuals with low serum 25(OH)D levels had a 63% increased odds of developing active TB [146]. A subsequent meta-analysis by the same group including data from seven studies showed significantly (48%) higher odds of developing TB in the vitamin D-deficient group [146]. The association between vitamin D deficiency and TB is thought to be bidirectional [146,147]. In the presence of overt granulomatous inflammation, the increase in circulating 1,25(OH)_2_D produced by activated macrophages and monocytes results in up-regulation of the expression of *CYP24A1* encoding the enzyme 25-hydroxyvitamin D-24-hydroxylase, which, in turn, converts 25(OH)D and 1,25(OH)_2_D into a water-soluble inactive carboxylic acids [2,6,148]. Moreover, serum 25(OH)D levels can be affected by anti-tuberculosis and concurrent medications such as antiretroviral drugs which are commonly used for treatment of comorbid HIV infection [2,149]. On the other hand, the association could be explained by the insufficient amount of 25(OH)D substrate required for the conversion into 1,25(OH)_2_D to stimulate granulomatous immune response against the invading organisms. 

To date, the efficacy of vitamin D supplementation as an adjunctive treatment for TB remains unclear as some RCTs demonstrated the impact of vitamin D on improving treatment outcomes such as smear conversion rate [150], culture conversion rate [151], time to sputum culture conversion [152], and improvement of clinical and radiographic findings [153], while others did not [154,155,156,157]. 

To sum up, vitamin D is essential for host inflammatory response to TB. Vitamin D deficiency is associated with an increased risk for developing active TB infection. However, whether supplementation of vitamin D can improve treatment outcomes for TB is still to be clarified due to differences in the results across the clinical trials.

### 5.7. Sepsis and Critical Illness

Sepsis, a systemic inflammatory host response to a microbial pathogen, is a major cause of death among hospitalized patients in the intensive care unit (ICU) [158]. Multiple observational studies reported the link between low level of serum 25(OH)D and the occurrence of sepsis, as well as increased morbidity, mortality, and prolonged length of stay in the ICU in septic and critically ill patients [159,160]. The relationship could be explained by the effects of 1,25(OH)_2_D which prevents overexpression of inflammatory cytokines and promotes anti-bacterial responses in innate immunity [1,5,161]. In addition, vitamin D_3_ and its metabolites can exert non-genomic actions on endothelial cells to prevent vascular leakage, which theoretically could be life-saving in septic shock [37]. It is also possible that low serum 25(OH)D levels in sepsis and critical illness could be caused by extravascular leakage of vitamin D-binding protein and increased 25-hydroxyvitamin D-24-hydroxylase activity due to systemic inflammation [162,163]. 

A number of RCTs have been conducted in order to investigate the impact of vitamin D on clinical and biochemical outcomes in sepsis and critically ill patients, yielding heterogeneous results. In a pilot clinical trial, giving a single enteral dose of 400,000 IUs of vitamin D_3_ to sepsis patients compared with placebo was shown to increase serum cathelicidin [164,165]. Whereas, the same effect was not observed when giving intravenous 2 µg of 1,25(OH)_2_D_3_ to patients with severe sepsis or septic shock [166]. A RCT gave enterally 540,000 IUs of vitamin D_3_ followed by monthly maintenance doses of 90,000 IU for 5 months to vitamin D-deficient, defined by serum 25(OH)D <20 ng/mL (50 nmol/L), or placebo to 475 critically ill patients and observed a significant decrease in hospital mortality in a subgroup of 200 patients with severe vitamin D deficiency (serum 25(OH)D <12 ng/mL/30 nmol/L, hazard ratio 0.56; 95% CI: 0.35–0.90) [167]. In a post-hoc analysis after excluding patients who died or were discharged within 7 days after study inclusion, vitamin D supplementation was associated with a decrease in 28-day mortality in the remaining 410 patients (odds ratio 0.58; 95% CI: 0.35–0.97) [168]. Another pilot study that gave a single dose of enteral 500,000 IUs or 250,000 IUs of vitamin D_3_ or placebo to 31 vitamin D-deficient mechanically ventilated ICU patients observed a decrease in hospital length of stay in the vitamin D groups compared to the placebo group [169]. Nevertheless, in a larger clinical trial that gave a single dose of enteral 540,000 IUs of vitamin D_3_ or placebo to 1360 critically ill patients, the impact of vitamin D_3_ administration on mortality and other clinical outcomes was not observed [170].

It can be concluded based on the current evidence that critically ill patients have a very high risk for vitamin D deficiency and therefore should be screened and treated for this condition. In fact, some studies have demonstrated a potential benefit on hospital outcomes in this group of patients. However, inpatient supplementation of vitamin D is still not universally accepted given the inconsistent results from clinical trials.

### 5.8. Respiratory Viral Infection and COVID-19

It is known that the outbreak of influenza infection is periodic and usually occurs during the wintertime at higher latitudes but is sporadic throughout the year in the tropical area [171]. One of the proposed explanations is that the seasonal outbreak could be due to a seasonal variation in circulating levels of 25(OH)D which reaches the lowest levels in the winter [172]. Several studies have supported this hypothesis as they reported the independent association between low level of serum 25(OH)D and incidence and severity of respiratory tract infection in children and adults [173,174]. A prospective cohort study in healthy adults living in New England showed a two-fold reduction in the risk of developing acute respiratory tract infection (ARI) in those with serum 25(OH)D levels of 38 ng/mL (95 nmol/L) or more [175]. A case-control study in children aged less than 2 years reported that children requiring hospitalization for ARI had significantly 1.7-times higher odds of vitamin D deficiency as compared to those with mild ARI [174]. This indicates the protective effects of sufficient vitamin D status against respiratory viral infection. Respiratory viruses enter the respiratory epithelium via the specific entry receptors where it causes cellular and tissue damages and triggers innate and adaptive immune responses, which then result in airway and systemic inflammation and, in severe cases, life-threatening sepsis or acute respiratory distress syndrome [176,177]. 1,25(OH)_2_D exerts anti-viral activities and modulates inflammatory response to viral infection by stimulating cathelicidin release, modulation of toll-like receptor expression and NK cells function, as well as suppressing overexpression of proinflammatory cytokines [178]. A recent meta-analysis of 25 RCTs showed that supplementation of vitamin D_2_ or D_3_ can protect against the development of acute respiratory tract infection compared with placebo (odds ratio 0.88; 95% CI: 0.81–0.96) [179]. 

The rise of the COVID-19 pandemic, the out-of-proportion rate of symptomatic infection, morbidity and mortality observed in African American and obese individuals suggests the possible impact of vitamin D on host response and susceptibility to the infection as obese and Black individuals are known to have an elevated risk for vitamin D deficiency [2,180,181]. Apart from the immunomodulatory and anti-viral effects, 1,25(OH)_2_D acts specifically as a modulator of the renin–angiotensin pathway and down-regulates the expression of angiotensin converting enzyme-2 expression, which serves as the host cell receptor that mediates infection by SARS-CoV-2 [182]. It is therefore proposed that supplementation of vitamin D can reduce the risk and severity of COVID-19 infection [183,184]. 

Although the efficacy of vitamin D is still unclear as the results of ongoing clinical trials are still pending, it is advisable that one should maintain adequate vitamin D intake to achieve the desirable serum 25(OH)D level of 40–60 ng/mL (100–150 nmol/L) in order to minimize the risk and severity of COVID-19 infection. It is well documented that worldwide on average approximately 40% of children and adults have circulating levels of 25(OH)D <20 ng/mL (50 nmol/L) and approximately 60% <30 ng/mL (75 nmol/L) [185]. Thus, patients presenting to the hospital with COVID-19 are likely to have vitamin D deficiency or insufficiency. It is therefore reasonable to institute as a standard of care to give at least one single dose of 50,000 of vitamin D to all COVID-19 patients as soon as possible after being hospitalized. For patients who are intubated and are being fed by a G-tube, they should be treated with a liquid form of vitamin D. Drisdol is a pediatric liquid vitamin D_2_ formulation that contains 8000 IUs per mL that can be given daily to these patients to treat vitamin D deficiency.

## 6. The Concept of Individual Responsiveness to Vitamin D

Despite the promising experimental data indicative of immunomodulatory effects of vitamin D supported by the observed association between low serum 25(OH)D level and multiple immune-related diseases, there is marked discrepancy in the results among clinical trials to determine the impact of vitamin D for treatment and prevention of most disorders. This could be explained by differences in dosage, form (vitamin D or 1,25(OH)_2_D or other metabolites and analogs), route of administration (oral, injection), patients characteristics including baseline levels of 25(OH)D, and outcome measurement. One of the major issues that could underpower the RCTs is that many of them were not truly controlled since control subjects were still permitted to take a certain amount of vitamin D up to a certain limit, usually 600 or 800 IUs daily [10]. It has been shown that even 600 IUs of vitamin D daily can have a significant effect on gene expression in immune cells [186].

**Figure 5:**
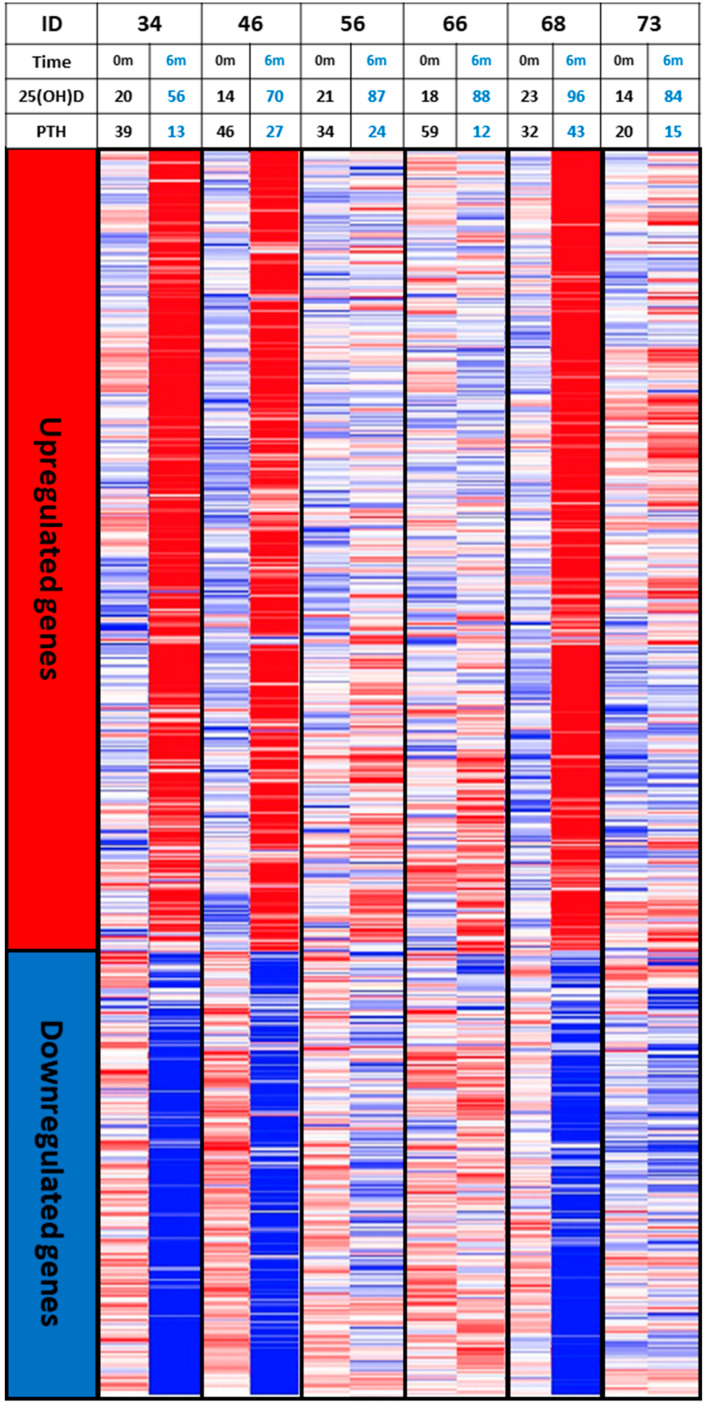
Heatmaps of vitamin D responsive genes whose expression response variation in 6 vitamin D-deficient subjects taking 10,000 international units per day of vitamin D_3_ for 6 months showing that 3 subjects had a robust response in gene expression compared to the other 3 subjects who had minimum to modest responses even though these subjects raised their blood levels of 25(OH)D in the same range of ~60–90 ng/mL (150–225 nmol/L). Abbreviation: 0m: 0 month; 6m: 6 months; 25(OH)D: 25-hydroxyvitamin D; PTH: parathyroid hormone. Reproduced with permission from Holick MF, copyright 2019.

Equally important if not more is the notion that some individuals might be able to benefit from vitamin D more or less than others as high inter-individual difference in broad gene expression in human peripheral blood mononuclear cells (PBMCs) in response to vitamin D supplementation has been documented [186,187,188]. Carlberg et al. gave daily 3200 IUs of vitamin D_3_ to 71 prediabetic patients for 5 months and found robust changes in total gene expression in PBMCs only in about half of the subjects [187]. In a more recent clinical trial by Shirvani et al. [186], healthy adults who were vitamin D deficient and who received this same dose of vitamin D and raised their blood levels of 25(OH)D to the same degree showed marked differences in the level of expression of the same genes. This is dramatically illustrated in Figure 5 which shows that 60% of the healthy vitamin D deficient adults who received 10,000 IUs daily for 6 months had a robust response in gene expression compared to the other 40% who had minimum to modest responses even though these subjects raised their blood levels of 25(OH)D in the same range of 60–90 ng/mL (150–225 nmol/L) [186]. In addition, different patterns of serum metabolomic profile were also observed between the subjects with robust and minimum to modest responses in gene expression [186,189]. It is therefore possible that the effect of vitamin D supplementation on health outcomes at the populational level is diluted due to the possibility that some individuals might benefit from vitamin D differently from others. This may help explain the null results reported by some large RCTs aiming to investigate effects of vitamin D supplementation on non-skeletal outcomes [190,191].

What is responsible for determining individual responsiveness to vitamin D supplementation is still undetermined and requires further investigation. However, there are some potential genetic variations in vitamin D related pathways that might play a role and should be put into perspective. First, it has been observed that certain polymorphisms in the vitamin D receptor (*VDR*) gene are associated with individual risk of cardiovascular diseases, cancer, and some autoimmune disorders independent of serum 25(OH)D levels [192,193,194,195,196]. Second, only tissues that express the megalin/cubilin complex such as the kidney are able to uptake DBP-bound-25(OH)D, while most tissues can utilize only the free level [197]. Genetic polymorphisms in the vitamin D-binding protein (DBP) (*GC*) gene was shown to influence the ratio between circulating total and free 25(OH)D [197]. Finally, it has been demonstrated that the variations of vitamin D responsive elements (VDRE) located in the promotor regions of VDR target genes could significantly affect the binding affinity to VDR–RXR complex, resulting in varying expression of the target gene in response to VDR signaling [112,113,198]. Thus, it is possible that individuals carrying different polymorphisms of *VDR* or *GC* genes among others or VDRE in the target genes may have varying genomic responses to the comparable levels of total serum 25(OH)D and therefore might benefit from vitamin D differently. 

## 7. Conclusions

Vitamin D plays an essential undisputed role in the maintenance of calcium, phosphate, and bone metabolism. There is compelling evidence that immune cells convert 25(OH)D to 1,25(OH)_2_D in an unregulated manner and are dependent on the circulating levels of 25(OH)D to be at least 30 ng/mL (75 nmol/L) [4,17,18]. Once a 1,25(OH)_2_D is produced, it acts in an autocrine and paracrine fashion to modulate the innate and adaptive immune systems. There is also some evidence that vitamin D itself may modulate immune function in a non-genomic manner by stabilizing endothelial membranes [36]. Most of the evidence, to date, suggests that maintenance of a healthy vitamin D status is important for modulating the body’s immune function. Low serum levels of 25(OH)D are associated with multiple immune-related diseases including autoimmune disorders and infectious diseases. There is less convincing evidence that vitamin D is an effective treatment strategy for autoimmune diseases and infectious diseases with a few exceptions documented in this review. Whether vitamin D therapy is effective as an adjunctive immunomodulatory agent for treatment of most diseases it is still controversial based on heterogeneous findings from the clinical trials.

National and international programs should be instituted to educate the public about the health benefits of vitamin D and policies to fortify commonly consumed foods with vitamin D to reduce the risk of vitamin D deficiency during pregnancy, childhood, and in young and middle-aged adults when autoimmune disorders are most prevalent. In addition, improvement in vitamin D status from birth until death may help reduce the risk of infectious diseases such as influenza and COVID-19 that can have devastating consequences especially for the elderly. However, more investigation is needed to determine who would most benefit from vitamin D, and how much vitamin D is required for its maximum health benefit based on their individual vitamin D responsive profile. It is also unknown whether giving 1,25(OH)_2_D_3_ or one of its analogs is a reasonable approach for treating autoimmune disorders and infectious diseases. Blood levels of 1,25(OH)_2_D_3_ are tightly controlled and for good reason, i.e., any significant increase in circulating levels of 1,25(OH)_2_D will result in an increase in intestinal calcium absorption and, when uncontrolled, this causes hypercalciuria and ultimately hypercalcemia. It is more likely that the endogenous production of 1,25(OH)_2_D in the immune cells including monocytes and macrophages is what is required for vitamin D to have its immunomodulatory functions. 

Although most of the biologic effects of vitamin D have been related to its active metabolite there continues to be intriguing evidence that vitamin D itself may have its own biologic actions independent of its metabolism. Our hunter gatherer forefathers likely maintained serum vitamin D levels in the range of 10–50 ng/mL (25–125 nmol/L). This is supported by the observation that Maasai herders and Hadza tribesmen maintained serum 25(OH)D in the range of 40–60 ng/mL (100–150 nmol/L) [12,13]. To maintain these blood levels, a person would require ingesting approximately 4000–6000 IUs daily. This would therefore maintain circulating levels of vitamin D in the range of 20–40 ng/mL (50–100 nmol/L). The observation that in vitro vitamin D_3_ was much more effective than either 25(OH)D_3_ or 1,25(OH)_2_D_3_ in stabilizing endothelial membranes thereby reducing inflammation may help explain the interesting clinical observations that extremely high doses of vitamin D have been effective in treating or at least reducing symptoms of some autoimmune disorders including psoriasis, vitiligo, and multiple sclerosis [37,85]. The observation that children with congenital autosomal recessive ichthyosis and epidermolytic ichthyosis had a dramatic improvement in their skin disease when treated with 60,000 IUs of vitamin D once a day for 10 days adds strength to the argument that vitamin D itself may have its own important role in the maintenance of good health [199]. There are still open questions that need to be further investigated in order to take full advantage of the effect of vitamin D on the immune system for clinical practice. The bottom line is that there is no downside to increasing our intake of vitamin D to maintain serum 25(OH)D at at least 30 ng/mL (75 nmol/L), and preferably at 40–60 ng/mL (100–150 nmol/L) to achieve optimal overall health benefits of vitamin D.

## Figures and Tables

**Table 1:** The recommended dosage for vitamin D intake in individuals who are at risk for vitamin D deficiency and dosage of vitamin therapy treatment for patients with vitamin D deficiency.

Age Group	For Individuals at Risk for Vitamin D Deficiency		Treatment for Patients with Vitamin D Deficiency
	Daily Requirement	Upper Limit	
0–1 years	400–1000 IU	2000 IU	- 2000 IU/d or 50,000 IU/wk of vitamin D_2_ or D_3_ for at least 6 wk to achieve serum 25(OH)D >30 ng/mL (75 nmol/L) - maintenance therapy of 400–1000 IU/d
1–18 years	600–1000 IU	4000 IU	- 2000 IU/d or 50,000 IU/wk of vitamin D_2_ or D_3_ for at least 6 wk to achieve serum 25(OH)D >30 ng/mL (75 nmol/L) - maintenance therapy of 600–1000 IU/d
>18 years	1500–2000 IU	10,000 IU	- 6000 IU/d or 50,000 IU/wk of vitamin D_2_ or D_3_ for 8 wk to achieve serum 25(OH)D >30 ng/mL (75 nmol/L) - maintenance therapy of 1500–2000 IU/d
Obese and malabsorptive patients	4000–6000 IU	10,000 IU	- Dosage should be increased by 2–3 times

Adapted from Evaluation, treatment, and prevention of vitamin D deficiency: an Endocrine Society Clinical Practice Guidelines [11].
